# Design of a New Built-in UHF Multi-Frequency Antenna Sensor for Partial Discharge Detection in High-Voltage Switchgears

**DOI:** 10.3390/s16081170

**Published:** 2016-07-26

**Authors:** Xiaoxing Zhang, Zheng Cheng, Yingang Gui

**Affiliations:** 1 State Key Laboratory of Power Transmission Equipment & System Security and New Technology, Chongqing University, Chongqing 400044, China; 20104300@cqu.edu.cn (Z.C.); yingang.gui@gmail.com (Y.G.); 2 School of Electrical Engineering, Wuhan University, Wuhan 430072, China

**Keywords:** ultrahigh frequency (UHF), high-voltage switchgear, partial discharge, Koch snowflake, multi-frequency

## Abstract

In this study a new built-in ultrahigh frequency (UHF) antenna sensor was designed and applied in a high-voltage switchgear for partial discharge (PD) detection. The casing of the switchgear was initially used as the ground plane of the antenna sensor, which integrated the sensor into the high-voltage switchgear. The Koch snowflake patch was adopted as the radiation patch of the antenna to overcome the disadvantages of common microstrip antennas, and the feed position and the dielectric layer thickness were simulated in detail. Simulation results show that the antenna sensor possessed four resonant points with good impedance matching from 300 MHz to 1000 MHz, and it also presented good multi-frequency performance in the entire working frequency band. PD detection experiments were conducted in the high-voltage switchgear, and the fabricated antenna sensor was effectively built into the high-voltage switchgear. In order to reflect the advantages of the built-in antenna sensor, another external UHF antenna sensor was used as a comparison to simultaneously detect PD. Experimental results demonstrated that the built-in antenna sensor possessed high detection sensitivity and strong anti-interference capacity, which ensured the practicability of the design. In addition, it had more high-voltage switchgear PD detection advantages than the external sensor.

## 1. Introduction

High-voltage switchgears, which are widely used in power systems, directly provide power to distribution networks and consumers. However, potential insulation defects are inevitable in electrical equipment, and high-voltage switchgears are more likely to produce insulation defects than other types of electrical equipment used in power systems because of their narrow internal space, complicated structure, and small insulation distance [1]. In many cases, insulation defects can result in uneven distribution of the electric field inside electrical equipment, especially serious electric field distortions at the defect position, which easily lead to partial discharge (PD) phenomena. Under the deterioration effect of persistent PD, sudden insulation faults may eventually occur. PD is thus considered a characteristic of insulation deterioration, therefore, PD detection has become a significant and feasible means to determine the insulation condition of electrical equipment [2].

Based on various physical and chemical phenomena occurring along with the PD, the main methods for PD detection can be classified into two categories: non-electric and electric measurements [3,4,5,6,7]. Non-electric measurements, which mainly refer to ultrasonic measurements [8,9], are generally used as an auxiliary detection method due to their low detection sensitivity. Electric measurements mainly include the transient earth voltage (TEV) [10], radio frequency (RF) [11] and ultrahigh frequency (UHF) methods [12]. Among these methods, the UHF one, which offers features of great anti-interference capability and high sensitivity as a noncontact measurement, has been extensively applied in PD detection. The UHF antenna sensors which are used to acquire the UHF signals caused by PD play the key role in UHF detection technology. According to the different installation locations of the sensors, UHF antenna sensors can be classified into built-in sensors and external sensors. Compared with the flexible and varied design of external sensors, the structures and sizes of built-in sensors are extremely limited in order to not affect the internal electrical equipment insulation environment. In general, built-in sensors have better PD detection performance than external sensors, but external antennas have other attractive characteristics such as the ability to monitor and locate PD over a much larger area. Currently, a variety of built-in and external UHF sensors with different performances and shapes have been designed, fabricated, and applied for PD detection in diverse electrical equipment such as transformers, and gas insulated switchgears (GIS) [13,14,15,16,17].

During field experiments, external UHF sensors are usually used to detect PD of high-voltage switchgears. However, their detection sensitivity and anti-interference capability are greatly limited because of the electromagnetic shielding effect of the all-metal switchgear casing and the electromagnetic interference from the surroundings. Furthermore, few specific built-in UHF sensors have been designed based on PD detection characteristics and the internal structure of high-voltage switchgears. In 2013, Yao designed a built-in UHF sensor for high-voltage switchgear PD detection [1], and practical application confirmed the effectiveness of the sensor. However, with a complex structure and a large volume, the sensor cannot be easily installed. It can also inevitably affect the internal electrical insulation environment when installed in the high-voltage switchgear, which could potentially lead to new insulation defects. Therefore, it is necessary to design a new specific built-in UHF sensor for high-voltage switchgear PD detection.

**Figure 1:**
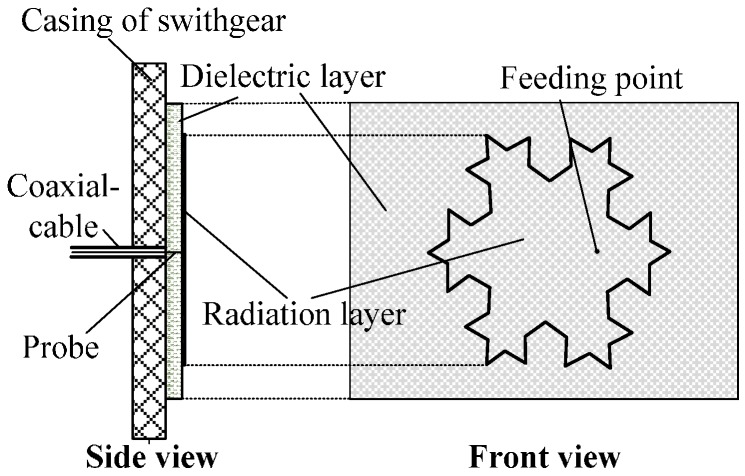
Structure of the built-in antenna sensor.

We observed the casings of high-voltage switchgear present large and flat features, although the high-voltage switchgear has a narrow interior space, complex components, and a compact structure. In this paper, a built-in UHF antenna sensor for high-voltage switchgear is designed, which adequately meets the high-voltage switchgear PD detection requirements. The casing of the high-voltage switchgear is initially used as the ground plane of the antenna sensor, and the antenna sensor is tightly attached to the inner wall of the high-voltage switchgear, which integrates the sensor into the switchgear. The antenna sensor diagram is shown in Figure 1. A microstrip antenna is adopted as a sensor prototype to achieve integration of the sensor and the switchgear. The microstrip antenna possesses a two-dimensional flat structure, which does not affect the internal electrical insulation environment. However, the narrow band of common microstrip antennas is a critical defect, and conventional methods for broadening the frequency band are inapplicable because of the internal space limitations in high-voltage switchgears, so the Koch snowflake patch is adopted as the radiation patch of the microstrip antenna, which can produce several resonant points to indirectly expand the antenna detection frequency band. As a result, it can greatly improve the PD detection performance of the sensor.

## 2. Basic Structure and Principle of the Antenna Sensor

### 2.1. The Formation of the Antenna Sensor

**Figure 2:**
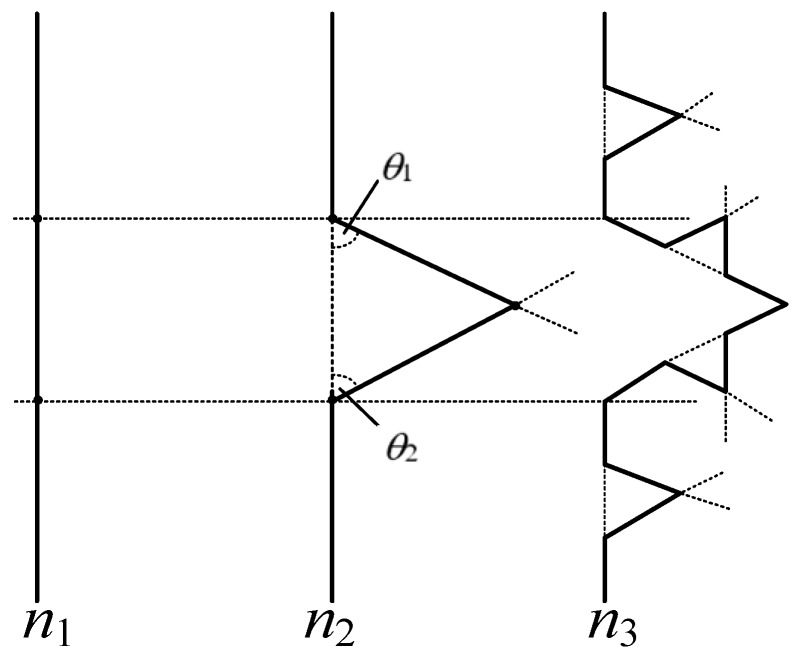
Iterative process of Koch curve.

In this study, the snowflake patch is selected as the radiation patch of the microstrip antenna. The snowflake patch, also known as Koch island-type patch, has a fractal structure consisting of three Koch curves. The Koch curve that resembles snowflakes is a type of classic fractal geometry structure and mathematical curve proposed by the Swedish mathematician Helge Von Koch in 1904 [18,19]. The iterative process of the Koch curve is shown in Figure 2.

**Figure 3:**
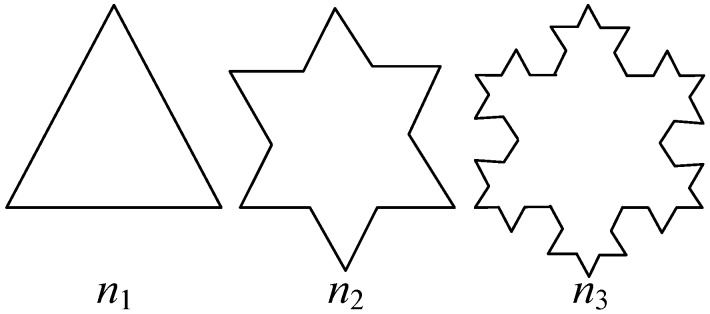
Iterative process of Koch snowflakes structure.

In this iterative process, line segment *n*_1_ is initially divided into three equal segments. Then, the middle line segment is removed and two new line segments are constructed through the two breakpoints, with the angles between the new line segments and the original segment being *θ*_1_ and *θ*_2_, respectively. The two segments intersect with each other to form a new curve *n*_2_. The same process is applied to each segment of curve *n*_2_ to obtain curve *n*_3_. In this fashion the higher-order Koch curve can be obtained. Considering the fact that *θ*_1_ and *θ*_2_ in the figure can be any value, the curve will have different shapes under different values of *θ*_1_ and *θ*_2_. In this study, *θ*_1_ and *θ*_2_ are both set to 60° to form the Koch snowflake structure. The iterative process is shown in Figure 3.

### 2.2. Theory Estimation of the Antenna Sensor Parameters

**Figure 4:**
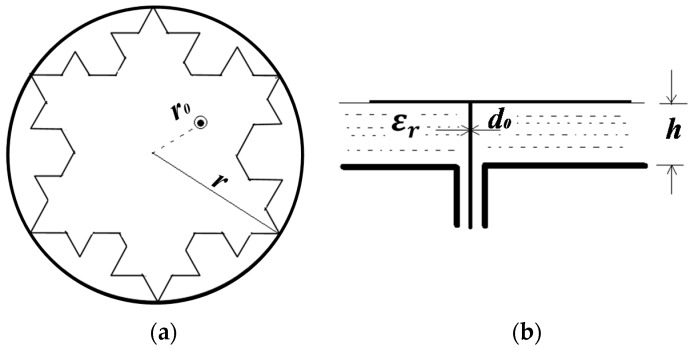
(**a**) Snowflake patch, (**b**) its circumcircle.

**Figure 5:**
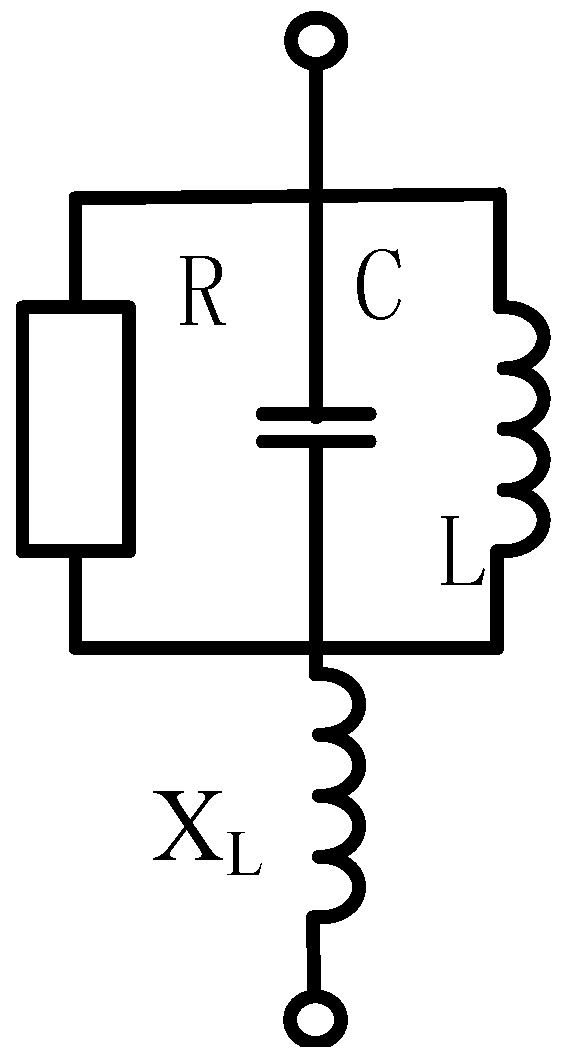
Equivalent circuit of the circular microstrip antenna.

The relevant parameters of the antenna sensor are estimated by calculating the parameters of its circumcircle microstrip antenna. Then, these parameters of the antenna are optimized with the aid of a electromagnetic simulation software named Ansoft HFSS (Ansoft Corporation, Pittsburgh, PA, USA). The snowflake patch and its circumcircle with radius *r* are illustrated in Figure 4. Coaxial probe-feed is adopted, and the radius of the feed position and the diameter of the feed probe are represented by *r*_0_ and *d*_0_, respectively, while *ε_r_* and *h* represent the relative dielectric constant and the dielectric layer thickness. The circular microstrip antenna is equivalent to an RLC resonant circuit (A circuit structure that is composed of resistance (R), inductor (L), and capacitance (C))according to circuit model analysis, as shown in Figure 5.

Considering the inductive reactance *X_L_* caused by the coaxial feed probe and the resonant resistance *R*, the input impedance *Z_in_* of the circuit can be defined as follows [20]:

(1) $$Z_{in}(f)=\frac{R}{1+Q_{T}^{2}{\left(\frac{f}{f_{s}}-\frac{f_{s}}{f}\right)}^{2}}-j[\frac{RQ_{T}(f/f_{s}-f_{s}/f)}{1+Q_{T}^{2}{\left(f/f_{s}-f_{s}/f\right)}^{2}}-X_{L}]$$

(2) $$X_{L}=\frac{377fh}{c}\ln(\frac{c}{\pi fd_{0}\sqrt{\varepsilon_{r}}})$$

(3) $$R=\frac{h^{2}E_{0}^{2}J_{1}^{2}(1.841r_{0}/r)}{2P_{T}}$$

where *Q_T_* is the quality factor of the resonance circuit, *f_s_* is the resonance frequency, *P_T_* is the total power loss, and *J_1_( )* is first-order Bessel function of the first kind.

The resonance frequency of the circular microstrip antenna can be approximated as follows:

(4) $$f_{s}=\frac{1.841c}{2\pi r\sqrt{\varepsilon_{r}}}$$

Based on Equations (1)–(4), the input impedance *Z_in_* of the antenna can be estimated. The reflection parameter *Γ* can be derived by using the Equation (5), where *Z_c_* is the system characteristic impedance. An impedance value of 50 Ohm is used for *Z_c_* in this study:

(5) $$Z_{in}=Z_{c}\frac{1+\Gamma }{1-\Gamma }$$

Eventually, the return loss *RL* and voltage standing wave ratio VSWR can be calculated using the following equations:

(6) $$RL=-20\log_{10}|\Gamma |,\;\mathrm{VSWR}\frac{1+|\Gamma |}{1-|\Gamma |}$$

## 3. Antenna Sensor Simulation and Parameter Optimization

The boundary of the antenna sensor is obtained via double Koch curve transformations of a 180 mm line segment, and the radius of its circumcircle is 103.9 mm. Based on Equation (4), the lowest resonant frequency of the antenna is approximately 403 MHz, which satisfies the PD detection need. A 280 mm long and 240 mm wide epoxy resin board with *ε_r_* equal to 4.4 is used as the dielectric layer. The dielectric layer thickness is initially set as 5 mm. A perfect conductor layer with 280 mm length and 240 mm width is used to simulate the high-voltage switchgear casing, and it is also regarded as the ground plane of the antenna sensor. The feed position and the dielectric layer thickness are simulated in details and optimized by taking the multi-frequency characteristic and impedance matching degree of resonant points into consideration.

### 3.1. Influence of the Feed Position on the Antenna Parameters

**Figure 6:**
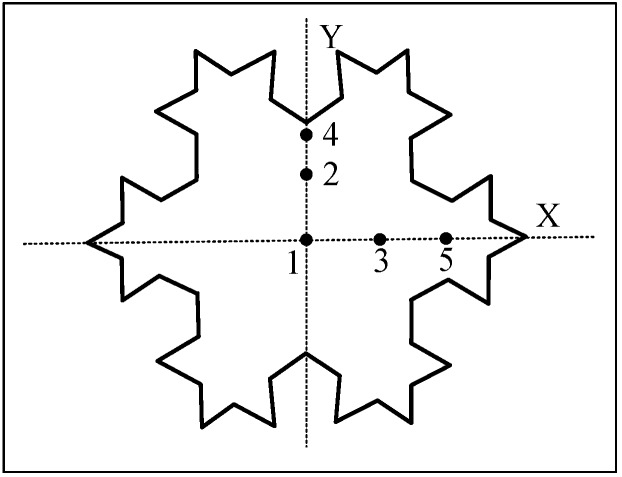
The antenna sensor.

Considering the symmetry of the antenna, feed points are selected on its symmetry axis. Figure 6 shows two symmetry axes of the antenna, denoted as the *X*-axis and *Y*-axis. Five points (Points 1–5) are selected to be the feed points, which are used to analyze the main resonant frequency and impedance matching degree at different feed positions. Points 3 and 5 along with Points 2 and 4 are respectively located on the *X*-axis and *Y*-axis. Point 1 is located at the geometric center of the antenna.

In addition, although the electromagnetic waves caused by the PD are distributed in the range of 300 MHz to 3 GHz, the main electromagnetic energy of the PD UHF signals concentrates under 1 GHz due to the significant attenuation of high-frequency signals. Therefore, the resonant points between 300 MHz and 1 GHz are considered in this study. The low frequency band mentioned below refers to this frequency band.

**Figure 7:**
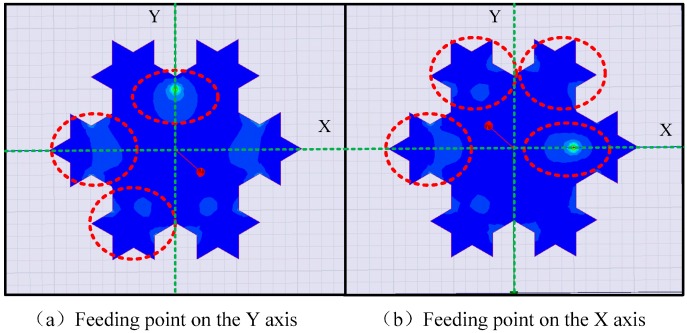
Current distribution of the antenna.

The simulation results, shown in Table 1, indicate that the antenna exhibits different resonant frequencies at different feed points, and these differences are embodied not only in the value of the resonant frequency but also in the number of resonant points. When the feed position is located at the geometric center of the antenna (Point 1), the antenna can only stimulate a single resonant point with a large resonant frequency. Because this point is situated at the core of the antenna, current paths in all directions are similar and singular. The simulation results of feed Points 2 and 4 on the *Y*-axis show that the antenna can stimulate three resonant points in the low-frequency band. By contrast, four resonant points can be stimulated when the feed points are Points 3 or 5 on the *X*-axis. By analyzing the current distribution of the antenna shown in Figure 7, it is found that the current path is more complicated when the feed point is located on the *X*-axis, which stimulates more resonant points.

Additionally, it also could be concluded from Table 1 that the resonant frequency at the minimum VSWR differs significantly as the feed point changes on the same axis, whereas the resonant frequency at diverse resonant points exhibits a slight change. According to the aforementioned simulation analysis, the feed position has a considerable influence on the resonant frequency and impedance matching degree at the resonant points. Thus, it is necessary to optimize the feed position of the antenna sensor. In this work, the optimal feed position will be selected from the *X*-axis.

### 3.2. Influence of the Dielectric Layer Thickness on the Antenna Parameters

Not only does the dielectric layer of the antenna play a physical supporting role for the antenna, but its parameters also have a great influence on the performance of the antenna. This study conducts a simulation to analyze the influence of the dielectric layer thickness on the antenna. In the simulation, the feed point is selected on point 5 of *X*-axis, as shown in Figure 6. Dielectric layer thicknesses of 1, 2, 3, 4, and 5 mm are selected, and the simulation results are shown in Table 2.

The results in Table 2 indicate that the increase of the dielectric layer thickness has no influence on the number of resonant points. The lowest resonant frequency have a small change, and it is approximately 450 MHz. The resonant frequencies of the second and third resonant points show an increasing trend as the dielectric layer thickness increases. By contrast, the resonant frequency of the fourth resonant point decreases with the increase of the dielectric layer thickness. However, the increase or decrease in the resonant frequency is not obvious.

The VSWR values displayed in Table 2 show that the dielectric layer thickness changes have a significant influence on the VSWR. The VSWR of the fourth resonant point decreases, whereas the VSWR of the other resonant points present an evident increasing trend with the increase of the dielectric layer thickness. The relevant equations mentioned in the Section 2 can be used to explain the results qualitatively. Based on Equation (2), the inductive reactance *X_L_*, the quality factor *Q_T_* and the resonant resistance *R* will change accordingly as the dielectric layer thickness changes. These changes of parameters above lead to the changes of the input impedance *Z_in_*, which eventually results in the VSWR change of the antenna. Moreover, Equation (4) also manifests that the resonant frequency is unaffected by the dielectric layer thickness.

Based on the simulation analysis of the antenna performance with different dielectric layer thicknesses, we come to the conclusion that thickness has an insignificant effect on the resonant frequency, but a serious effect on the impedance matching degree. For these reasons, optimizing the dielectric layer thickness is very important to improve the performance of the antenna sensor.

### 3.3. The Antenna Performance after Optimization and Measurement

The above simulations show that the resonant points of the antenna and its VSWR are both influenced by the feed position and dielectric layer thickness, and the antenna possesses four resonant points when the probe is located at the *X*-axis. Thanks to the optimetrics of the simulation software, this study changes the probe position at *X*-axis and dielectric layer thickness to examine antenna parameters. The probe position is varied from 0 mm to 103 mm along the *X*-axis with a step of 1 mm, and the thickness is set from 0 mm to 5 mm with a step of 0.1 mm. With each change in position or thickness, a different VSWR will be obtained. Finally based on these VSWR values, the optimal parameter combination could be determined. The final design parameters are shown at in Table 3.

**Figure 8:**
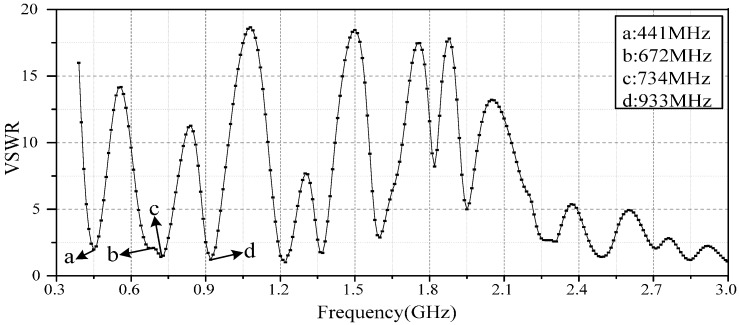
The simulated VSWR of the antenna.

Figure 8 shows the final simulated VSWR of the antenna sensor after parameter optimization. The simulation results show that the antenna sensor possesses four resonant points, with the lowest resonant point being 441 MHz, which is slightly different from the 403 MHz results obtained by the theoretical estimation. On the one hand, structural differences exist between the snowflake patch and circular patch. On the other hand, for the circular patch antenna, a certain difference is observed between the practical radius and the theoretical radius [19]. Considering these two factors, the practical resonant frequency is slightly higher than the theoretical resonant frequency. The other three resonant points at the low-frequency band are 672, 734, and 933 MHz. The VSWR of the four resonant points are 1.988, 2.102, 1.507, and 1.205, which indicates that the antenna possesses a good impedance matching degree. The results also show that the antenna presents good multi-frequency performance in the high-frequency range, and it can further enhance the PD detection ability of the sensor.

**Figure 9:**
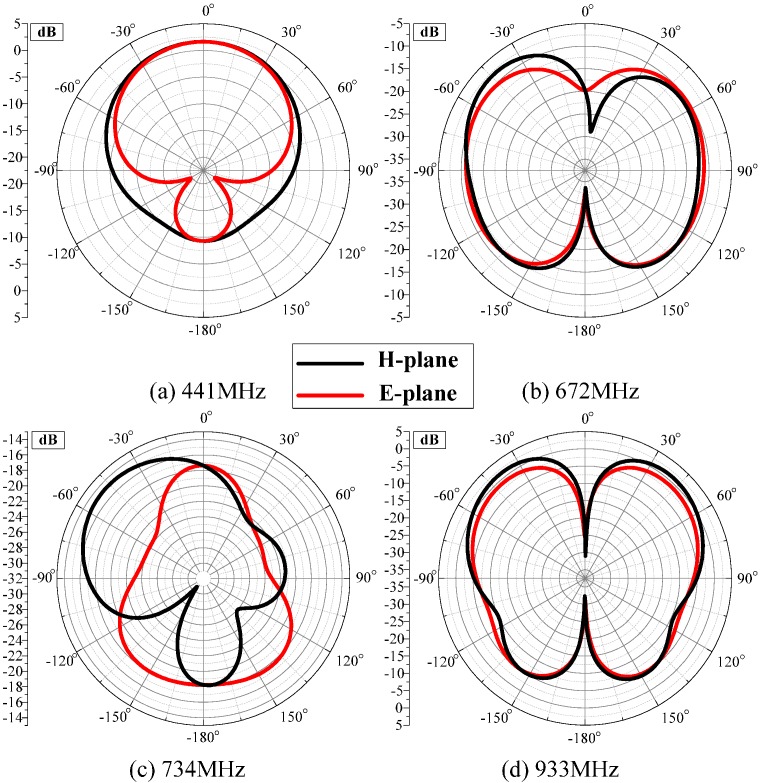
The antenna simulated radiation pattern in resonance points.

The ability to receive signals from different directions is important for the built-in antenna sensor, because the antenna sensor is tightly attached to the inner wall of the high-voltage switchgear. Therefore, the radiation direction of the four resonant points is analyzed in this study. Figure 9 shows the E-plane and H-plane antenna radiation patterns for four resonance frequencies.

When the frequency is 441 MHz, the optimal signal receiving direction is perpendicular to the radiation surface inside the switchgear, which shields external signals to some extent. That is to say, the capability of the antenna against external low-frequency interference is strengthened. Comparing the radiation patterns at different resonant frequencies of 441, 672, 734, and 933 MHz, it can be concluded that optimal receiving directions varies with different resonant frequencies, because the designed antenna sensor is a narrow-band antenna and four resonance frequencies differ considerably. Beneficially, all of them are located inside the switchgear. Therefore, the antenna sensor can effectively receive the PDs in all directions.

**Figure 10:**
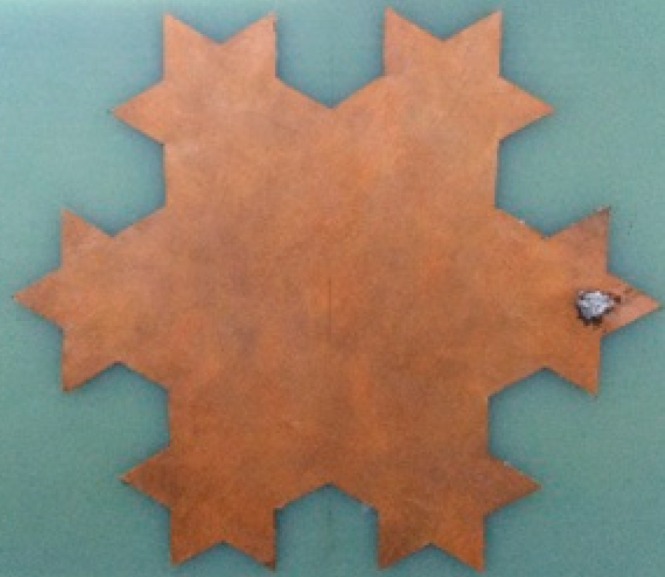
Physical map of the designed antenna.

**Figure 11:**
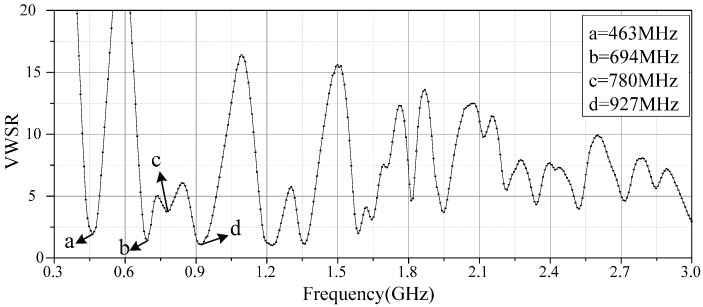
The measured VSWR of the antenna.

Next a built-in antenna sensor prototype is fabricated (Figure 10), and its VSWR is measured by a vector network analyzer (Anritsu 37369D, Kanagawa, Japan; frequency range from 20 MHz to 40 GHz) to verify the accuracy of the antenna sensor simulation results. The measured VSWR is shown in Figure 11, and the results indicate that the antenna sensor presents good multi-frequency performance. The four resonant points in the low-frequency band are 463, 694, 780, and 927 MHz, and their corresponding VSWRs are 1.911, 1.419, 3.754 and 1.122, respectively. This result also shows that the antenna possesses multiple resonant points from 1 GHz to 3 GHz. Therefore, it can be concluded that the measurement results basically accord with the simulation results.

Although the single resonant point shows a narrow frequency band because of the limitations of the characteristics of the microstrip antenna, the PD detection ability of the antenna sensor can be indirectly strengthened because numerous resonant points exist at the same time. As far as PD detection is concerned, the antenna sensor with multi-frequency performance can detect more characteristic PD information, which is of great benefit to identify different insulation defects.

## 4. Measurement and Analysis of PD

### 4.1. PD Detection Experiment Platform

**Figure 12:**
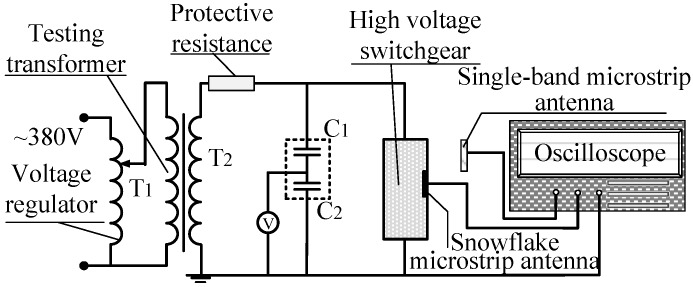
Experimental circuit diagram.

**Figure 13:**
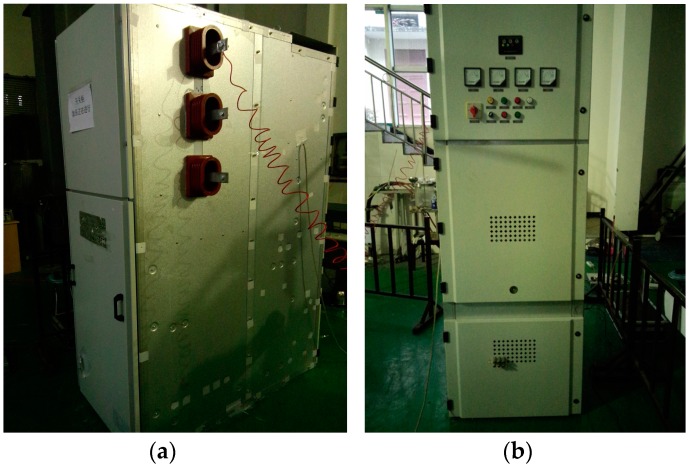
The high-voltage switchgear setup. (**a**) side and (**b**) front view.

PD detection experiments were conducted in a high-voltage switchgear unit to assess the actual PD detection capability of the built-in antenna sensor. The experiment circuit is shown in Figure 12. The built-in antenna sensor is tightly attached to the inner wall of the high-voltage switchgear. An external UHF antenna sensor is simultaneously adopted to verify the superiority of the built-in antenna sensor for PD detection. The external UHF antenna sensor is a single-band microstrip patch antenna [21] (center frequency: 390 MHz, frequency band: 350–450 MHz), which is placed at the door gap of the switchgear. In order to collect PD signals better and avoid the influence of the sensor position on experimental results, the external UHF antenna sensor remains stationary and as close to the insulation defects as possible. The 10 kV three-phase high-voltage switchgear shown in Figure 13 is adopted in this experiment. It has five isolation spaces: bus, circuit breaker, cable, secondary instrument and standby isolation space. The experiments are conducted in a cable isolation room because of its relatively high fault rate in actual operation. An AC testing transformer (YDTW-25/100, Tang Longsheng Electric Co., LTD, Wuhan, China) is used as experiment power source, and a high-speed oscilloscope (LeCroy Oscilloscope, New York, NY, USA; its highest sampling rate is 20 GHz) is used to record the waveform of PDs. The needle-to-plate model, with 10 mm separation distance, is used as the insulation defect to generate PD, and the voltage applied to the needle-to-plate is evenly boosted with a gradient of 0.5 kV.

### 4.2. PDs of a Power Frequency Cycle

**Figure 14:**
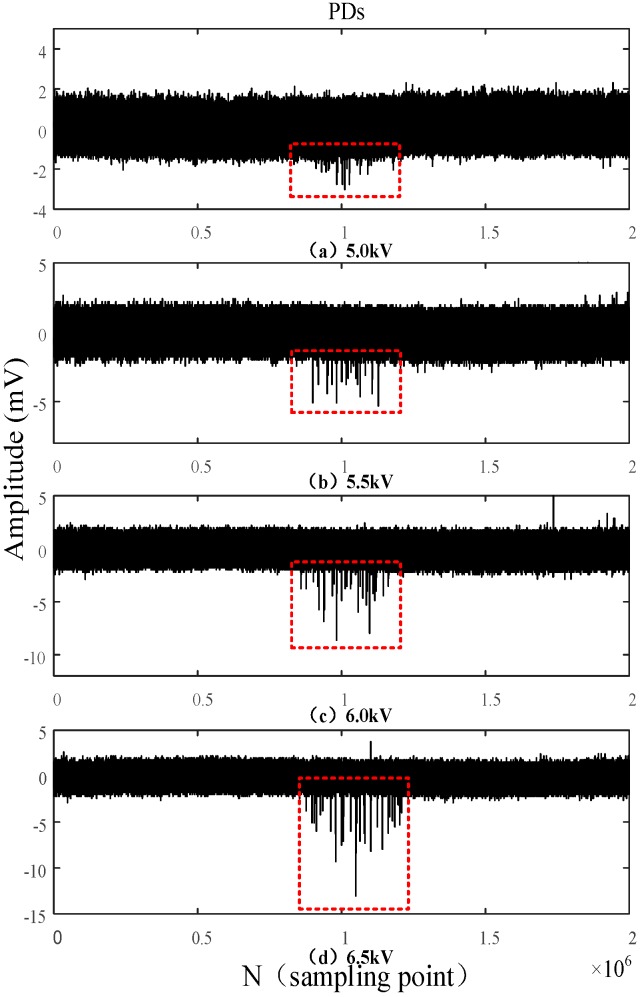
PDs detected by the built-in antenna under different voltages.

**Figure 15:**
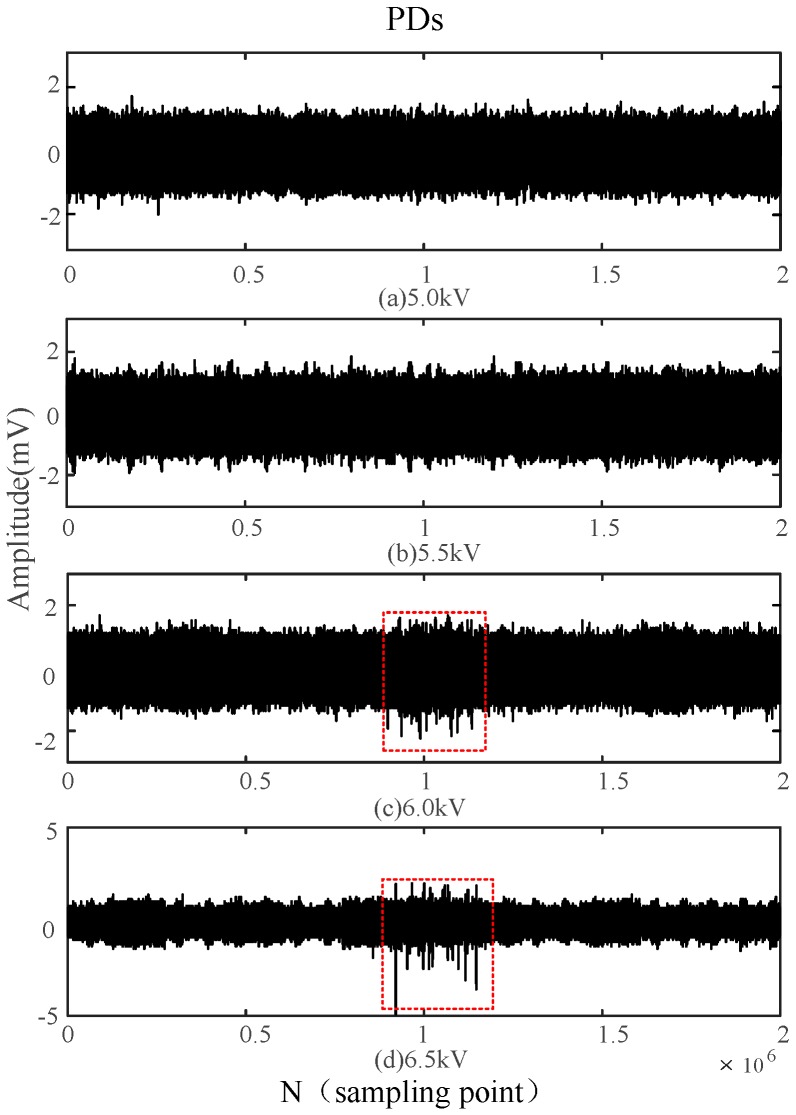
PDs detected by the external antenna under different voltages.

In order to assess the detection performance of the built-in sensor and verify its superiority, the PDs with a power-frequency cycle (20 ms) are analyzed under different voltages, with a sampling frequency of 100 MHz. Figure 14 and Figure 15 show the waveforms of PDs detected by the built-in antenna and the external antenna for different voltages, respectively.

The built-in sensor is able to detect a small discharge when the voltage is only 5 kV, whereas the initial discharge is only detected by external sensor when the voltage reaches 6 kV. When the voltage is set to 5.5 kV, signals detected by the built-in sensor indicate there is a continuous and evident discharge in the high-voltage switchgear. However, the external sensor only detects continuous and evident PDs when the voltage is increased to 6.5 kV. Those experimental results show that the designed built-in sensor possesses good detection sensitivity, and it detect insulation defects earlier than the external sensor.

The waveforms of PDs shown in Figure 14 and Figure 15 indicate that the PDs detected by the external sensor possess more burrs, from which we can conclude that the external sensor is seriously affected by the external electromagnetic interference. Compared with the external sensor, the built-in sensor shows a better anti-interference capability because of the electromagnetic shielding effect of the all-metal casing.

**Figure 16:**
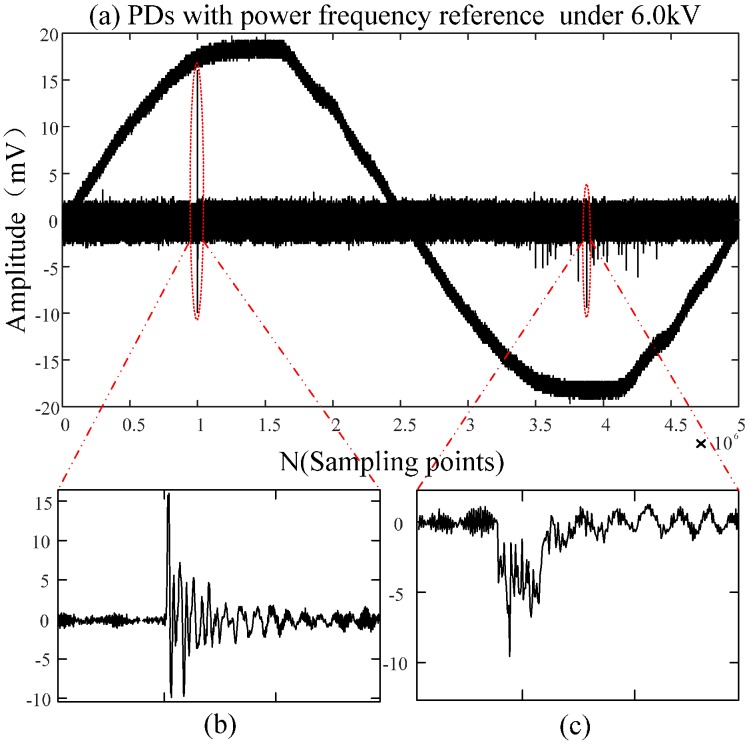
(**a**) PDs with frequency reference under 6.0 kV; (**b**) The enlarged view of PDs occurring in the positive half-period; (**c**) The enlarged view of PDs occurring in the negative half-period.

By analyzing the PDs detected by the built-in sensor under different voltages as shown in Figure 14, it can be concluded that the discharge times and the amplitude of PDs will increase as the applied voltage increases. Those discharges occur intensively in the negative half-period of the power-frequency cycle, because a positive voltage is applied to the needle electrode. The corona onset voltage of the negative half-period is less than that of the positive half-period because of the polarity effect. Partial and continuous discharges in the negative half-period are firstly observed when the applied voltage is less than the breakdown voltage. In order to verify the correctness of the above analysis, the applied voltage is sampled as the power-frequency reference for PDs, but gathering the integral PDs with low sampling frequency is challenging because the PD in the positive half-period is less and not continuous. Therefore, sampling frequency is set as 250 MHz, and the experimental results are consistent with the above analysis as shown in Figure 16. Figure 16b,c are an enlarged view of PDs occurring in the positive half-period and negative half-period, respectively. The information indicates that PDs occurring in the positive half-period possesses a larger amplitude, and the maximum amplitude is positive, while the maximum value of PDs occurring in the negative half-period is negative. Moreover, there are evident differences in the waveform of PDs occurring in the different half-periods. Subsequently, the single PDs occurring in the positive half-period are acquired and verified based on the previously presented characteristics.

### 4.3. The Single PDs Occurring in the Positive Half-Period

**Figure 17:**
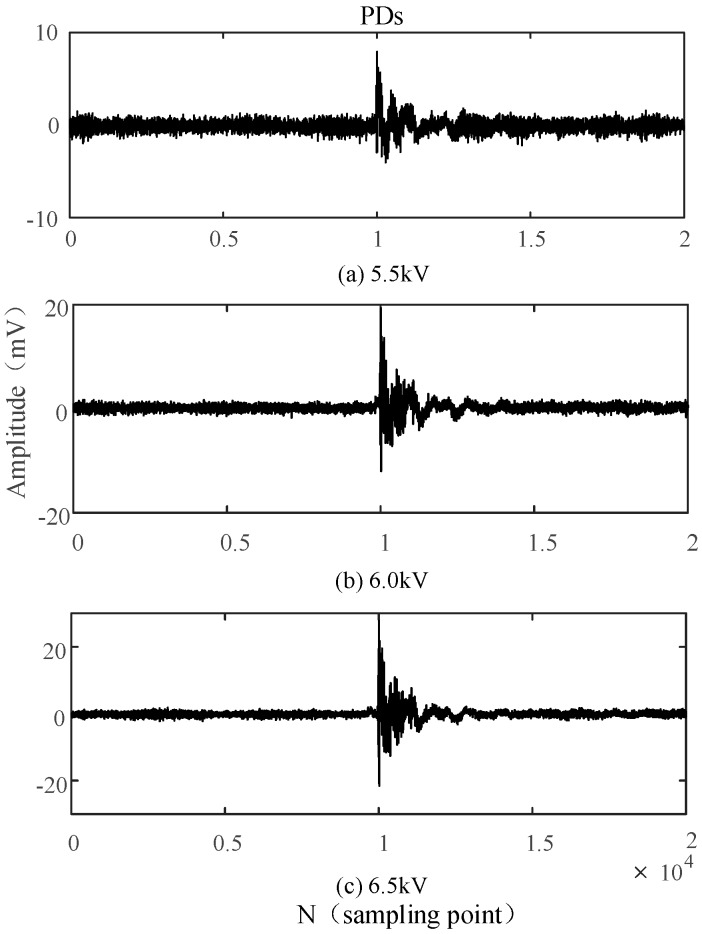
Single PDs detected by the built-in antenna under different voltages.

**Figure 18:**
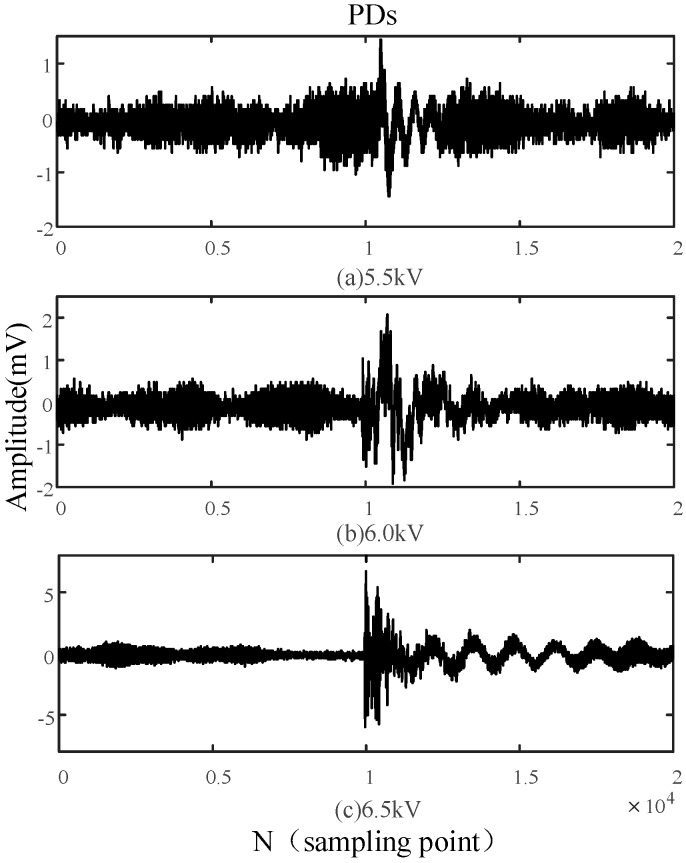
Single PDs detected by the external antenna under different voltages.

Due to the polarity effect of the needle-to-plate defect, insulation breakdown frequently occurs in the positive half-period. Therefore, it is an indispensable capacity for an UHF antenna sensor to detect single PDs occurring in this half-period. In this paper, single PDs (2 ms) are sampled and analyzed under different voltages, with a sampling frequency of 10 GHz, in order to further assess the PD detection capability of the built-in sensor. Figure 17 and Figure 18 show the single PDs detected by the built-in antenna sensor and the external antenna sensor under different voltages, respectively.

Figure 17 indicates that the amplitude of PDs in the positive half-period are significantly larger than that of PDs in the negative half-period under the same voltage conditions, and the magnitude of the PD amplitude increase is more obvious under the same conditions. Compared with the single PDs detected by the external antenna as shown in Figure 18, not only do PDs detected by the built-in sensor possess a larger amplitude under the same voltage conditions, but the amplitude variation of PDs is also more evident for the same voltage difference. These experimental results also show that the built-in antenna sensor possesses high detection sensitivity and strong anti-interference ability, and the external antenna sensor presents poor PD detection capacity because of the electromagnetic shielding effect of the all-metal casing and the interference from the electromagnetic environment.

In general, parameters used to characterize the strength of PD in high-voltage switchgear, namely the severity of the internal insulation defects, are closely related to the times and amplitudes of PDs. They all can be detected through the built-in antenna sensor presented in this paper. The experimental results show that the built-in antenna sensor has great PD detection capacity, and it has more advantages in high-voltage switchgear PD detection than the external sensor.

## 5. Conclusions

A new built-in UHF antenna sensor that adequately incorporates the structural characteristics of switchgear is designed and applied in a high-voltage switchgear unit. The casing of the switchgear is initially used as the ground plane of the antenna sensor, which integrates the antenna sensor into the high-voltage switchgear. The two-dimensional plane structure of the microstrip antenna does not affect the internal operation environment of the high-voltage switchgear, while the antenna presents a relatively high anti-interference capacity because of the electromagnetic shielding effect of the all-metal casing. The feed position and the dielectric layer thickness are simulated to analyze the multi-frequency performance of the sensor. The built-in antenna sensor possesses four resonant points with good impedance matching after optimization, which results in good multi-frequency characteristics in the entire working frequency band. A built-in antenna sensor prototype is fabricated based on a sensor simulation model, and the actual measurement results show that the antenna presents favorable multiple-frequency performance, which accord with the simulation results. PD detection experiments are conducted in the high-voltage switchgear, and an external UHF antenna sensor is used as a contrast to assess the PD detection ability of the built-in antenna sensor. The experimental results demonstrate that the built-in antenna sensor possesses high detection sensitivity and strong anti-interference capacity, which proved the validity and practicability of the sensor designed in this study, and built-in sensor has more advantages at the high-voltage switchgear PD detection than the external sensor.

## Figures and Tables

**Table 1:** Resonant points and minimum standing wave ratio at different feed position.

Feed Point	Resonant Frequency (MHz)	Minimum VSWR
1	935	2.876 (935 MHz)
2	446, 658, 928	1.411 (928 MHz)
3	444, 656, 734, 919	1.235 (656 MHz)
4	446, 657, 921	1.552 (657 MHz)
5	454, 674, 728, 922	1.308 (922 MHz)

**Table 2:** Resonance points and their VSWR under different dielectric layer thickness.

Thickness (mm)	Resonance Frequency (MHz)		VSWR
1	1	450	1.126
	2	644	1.344
	3	711	1.383
	4	940	3.167
2	1	453	1.685
	2	654	2.515
	3	723	1.389
	4	940	1.906
3	1	452	2.240
	2	660	3.494
	3	728	1.964
	4	934	1.523
4	1	453	2.660
	2	666	4.158
	3	730	2.491
	4	929	1.402
5	1	454	2.977
	2	674	4.559
	3	728	2.887
	4	922	1.308

**Table 3:** Final design parameters.

Length (mm)	Width (mm)	Base Koch Curve (mm)	Thickness (mm)	Probe Position (mm)
280	240	180	5	(83, 0)
